# Journey mapping as an inclusive research tool: Capturing the learning journeys of health professions educators with dyslexia

**DOI:** 10.1111/medu.70104

**Published:** 2025-11-18

**Authors:** Sarah McLaughlin, Asim Ali, Steve Jennings

**Affiliations:** ^1^ University of Bristol Medical School Bristol UK

## Abstract

**Introduction:**

The field of health professions education has seen growing emphasis on inclusive pedagogies and learner diversity. Universal Design for Learning (UDL) offers a framework for designing educational experiences that accommodate diverse learning needs. Applying this principle to research, we must consider not only *what* we research but also *how* we research. Dyslexia is one example where traditional research practices may unintentionally marginalise participant voices. Conventional research interviews—especially those relying heavily on verbal recall and heavy question–answer formats—may not be the most accessible or inclusive method for gathering rich data with participants who are dyslexic. However, recent research reports that those with dyslexia excel at visualisation, creative thinking, identifying patterns and oral communication. In response to these characteristics and challenges, we adopted journey mapping to create a more inclusive research experience.

**Methods:**

We explored the learning journeys of health professions educators who have dyslexia. We conducted six semi‐structured online interviews utilising participant‐created journey maps as a creative and inclusive method for data construction. The maps were used as a visual prompt and a reflection tool before and during the interviews. Data were analysed using reflexive thematic analysis.

**Results:**

The mapping provided participants with an enjoyable, engaging, autonomous means of reflection, enabling them to structure and tell their stories in a personally meaningful way. It supported verbal articulation during interviews by offering visual scaffolding and helped reduce cognitive load by allowing time to reflect, plan and organise responses in advance.

**Discussion:**

Adapting research methods to ensure a more inclusive approach has the potential to create more authentic, rich data and a fun and engaging experience for participants. The visual nature of mapping is a key advantage; however, researchers must mitigate the potential harm to participants elicited through the depth of reflection of negative experiences. Clear instructions and reassurance relating to creative confidence are key to executing this approach.

## Abstract 2

Looking for a new creative and inclusive research method to access experience? Journey mapping could be for you.

## 1 INTRODUCTION

The field of health professions education has seen growing emphasis on inclusive pedagogies and learner diversity.^1^ Universal Design for Learning (UDL) offers a framework for designing educational experiences that accommodate diverse learning needs. When it comes to researching health professions education, this imperative must also extend to research methodologies.

Qualitative interviews are commonly used in education research as a straightforward method to explore participants' stories and experiences and generate nuanced and analytically robust data.^2^, ^3^ However, the richness of this data may be compromised if research approaches are not inclusive of all participants.^4^ Dyslexia, affecting approximately 10% of the UK population,^5^ is one example where traditional research practices may unintentionally marginalise participant voices.

Dyslexia is a ‘genetic difference in an individual's ability to learn and process information’.^6^ It is frequently described as a neurodevelopmental learning difficulty^7^ and is commonly associated with difficulties in reading and writing. Dyslexia affects various cognitive processes, such as working memory and processing speed. These cognitive processes are particularly relevant in research interview settings.^8^, ^9^

We conducted a study exploring the learning journeys of health professions educators with dyslexia. It was imperative that data collection tools led to authentic and credible understanding of participants' stories, and the meanings they gave to them, without constraint or discomfort. Considering the impact of dyslexia extends beyond literacy and can influence broader cognitive processing, including working memory, processing speed and verbal fluency,^7^, ^8^ we recognised the potential limitations of purely verbal, time‐constrained interviews.

Following Nind's^10^ conceptualisation of inclusive research as a process of praxis, phronesis and participant‐driven knowledge generation, our methodological choices considered participants' diversity and preferred modes of meaning‐making. Journey mapping provided a creative, participant‐led method that foregrounded their lived experiences and ways of knowing, aligning with inclusive research's ethical imperative to do research ‘with’ rather than ‘on’ participants. This paper draws on empirical data to offer practical insights into journey mapping as an inclusive method.

### 1.1 Understanding dyslexia in the context of research interviews

Dyslexia accounts for around 80% of reported learning difficulties in the United Kingdom,^11^ with rising numbers of higher education students self‐identifying as dyslexic.^12^ Rates are prevalent among health care professionals, with up to 10% of doctors in training affected.^13^, ^14^ Yet, research still frames dyslexia primarily as a childhood issue.

Although research on supporting neurodivergent health professionals is emerging, studies focused specifically on dyslexia remain limited.^1^, ^11^ This gap is concerning because these trainee educators will be the teachers and role models to our future health care professionals. Moreover, occupational potential may be hindered if trainee educators with dyslexia are not supported.^15^

We considered the use of interviews as a tool for our research project because we sought to gather rich insights into participants' learning journeys. We aimed to use methods that allowed participants to share their stories in ways that accommodated the cognitive challenges of dyslexia, which could otherwise hinder their participation. Key challenges for adults with dyslexia include the following^7^, ^8^:

**Working memory**—holding and manipulating information in real time, which is crucial during interviews when recalling details or responding to complex questions.
**Processing speed**—taking longer to decode and respond to verbal information, which could lead to anxiety or perceived underperformance in time‐pressured or highly verbal settings.
**Word retrieval and verbal articulation**—struggling to find the right words or express themselves clearly, especially under stress, which could hinder their ability to fully communicate their experiences during standard interviews.

These challenges suggest that conventional, verbally intensive interviews may not be the most inclusive approach to gather their experiences.

Despite the difficulties presented by dyslexia, recent studies indicate individuals with dyslexia are above average at visualising, mapping out complex concepts, oral communication, connecting with others and expressing themselves. They ‘see the world differently’ and are highly creative.^6^ In response to these characteristics and challenges, we turned to creative approaches with the aim of creating a more inclusive research experience.

### 1.2 Inclusive and creative methods

Inclusivity in education research relates not only to what we research but also to how we research. Inclusive research considers the research question; data collection and dissemination are accessible to participants.^16^, ^17^ Selecting appropriate methods that ensure participants are reciprocally engaged in their creation of data can facilitate increased participant authority in the research process and enhance trust and meaningful, accountable engagement.^18^, ^19^

Creative methods are an approach to data collection, creation and analysis, which involves participants spending time creating something symbolic or metaphorical using art‐based, or visual tools, which they subsequently reflect upon.^20^ Examples include photovoice, storytelling,^21^ drawing^22^ and Play‐Doh modelling.^23^ Such approaches may also be adopted by those problematising research methods with the intention of adapting their tools to afford a more inclusive research study.

Journey mapping is a creative method that uses participant‐created visual timelines to represent key life events, representing their meanings in some kind of chronological order.^24^, ^25^ They have been found to enhance inquiry by illuminating participant perspectives,^26^ supporting rich accounts of student experience,^27^ highlighting the diverse ways meaningful participation unfolds.^28^ Our study utilised journey mapping alongside semi‐structured interviews as a more inclusive approach to data creation. This paper will now outline the study design and present methodological insights.

## 2 METHODS

The study was guided by the research question ‘What are the unique learning experiences of UK health professions educators with dyslexia during their post graduate teacher training journeys?’ The research was underpinned by constructivist epistemology and symbolic interactionism. This lens sees individual behaviour, cognition and identity as being shaped by social interaction, and interaction shaping self‐concept and identity.^29^, ^30^

The study recruited six participants currently enrolled in a post graduate certificate (year one) or diploma (year two) health professions education (teacher training) programme at the end of the 2024/25 academic year. A purposive sampling approach was taken. All participants had a formal diagnosis of dyslexia (coincidently all were diagnosed as adults before or during their undergraduate studies), and English was their first language. They were qualified health professionals with teaching roles. They represented 4.3% of the course's student population that year. Recruitment involved a classroom poster, a PowerPoint slide in the final session, and an email to current and recent students (past 3 years). Interested participants received an information sheet and consent form. Ethical approval was granted by the University's Research Ethics Committee (Table 1).

**TABLE 1:** Participant information.

Pseudonym	Level of post graduate teacher training study	Role
Rachel	Diploma	Clinical Teaching Fellow (CTF)
Bob	Diploma	Psychiatrist
Peter	Certificate	CTF
Bella	Certificate	CTF
Blanche	Diploma	Physiotherapist
Gail	Diploma	Academic Clinical Fellow

Step 1—Pre‐interview meetings provided project details, answered questions and explained the rationale for creating the maps. Upon consent, participants were invited to create a learning journey map before their scheduled online interview. Instructions were broad to encourage creative freedom. Participants were advised to begin their maps as far back as they felt relevant, reflecting on key educational experiences, influences on their professional identity, teaching practices, their decision to commence the teacher training programme, and their learning during the programme.

**FIGURE 1:**
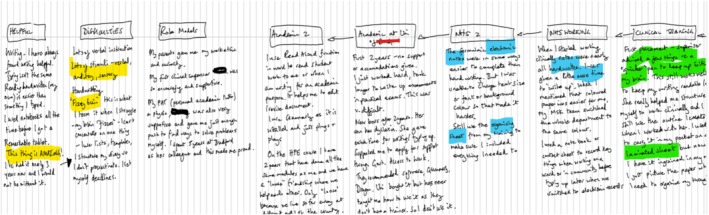
Example map. [Color figure can be viewed at wileyonlinelibrary.com]

Participants were reassured that no artistic skill was needed, addressing concerns about creative confidence.^31^ To support working memory and processing speed, the interview guide was shared 3 weeks in advance. The question prompts related to how they defined their dyslexia, how dyslexia impacted their educational journeys, and their practice as educators. This helped shape their reflections and maps (see Figure 1 for an example).

Step 2—Semi‐structured one‐to‐one interviews were carried out by the first author, lasting between 30 and 60 minutes, via Microsoft Teams. Participants were asked to explain what their maps were depicting, and an interview topic guide was used to further prompt discussions. Prompt questions related to how participants defined dyslexia, poignant moments during their educational journeys (from primary school to post‐graduate teacher training), experiences of being a health professions educator with dyslexia, and how they found creating the maps and taking part in the study.

Step 3—Data were analysed using Braun and Clarke's^32^ six‐step reflexive thematic analysis. Transcripts were printed in a different colour per participant. Authors one and three rewatched interviews together, pausing systematically throughout each to discuss key moments and code transcripts while reflecting on researcher positionalities. Author two contributed to the discussion and interpretation of codes and the creation of code labels. The maps themselves were not coded; however, participants' explanations and reflections created the data to be analysed. Transcripts were manually coded by cutting out words and phrases, which were discussed to construct code labels. Categories and, subsequently, themes were then developed in response to the research questions. Category titles were written on large paper, with codes visually arranged beneath. Pseudonyms matched transcript colours (e.g. blue = Bob, red = Rachel) to maintain anonymity while aiding organisation.

## 3 RESULTS AND DISCUSSION

The results section is presented as four ‘pearls of wisdom’ gleaned from our experience of using journey mapping. Our contribution argues that (1) mapping can be a fun and engaging tool, (2) its visual nature is a key advantage, (3) clear instructions for participation are vital, and (4) researchers should be prepared for the elicitation of significant emotions.

1 ‘It's quite fun actually’—Mapping can be a fun and engaging tool

Consistent with Nind's^10^ principle of respecting participants' ways of knowing and expressing themselves, the method encouraged an open reflection upon the elements participants' felt were salient. Overall participants expressed positive experiences—‘It's quite an interesting exercise to do’ (Bob). The opportunity to reflect upon their journeys was valued and to have their stories heard was also appreciated ‐

Oh, it's quite fun actually. This is great. I just get to talk about myself for 40 minutes. (Bella)

The task provided the space for participants to engage on their own terms, in their own time. There were expressions of relief at being able to ‘prepare my thoughts’ (Peter) rather than ‘panic about saying it all at once’ (Blanche), as evidenced by the range of different maps created by individuals.

The maps anchored participants in specific memories and experiences. The opportunity to reflect and be heard also provided some recognition and validation of their hard work, as Gail reflected ‘but I did get through uni, and I survived’. Also, the achievements in their educational journeys:

Yeah, it was a bit fun, kind of looking back on school and stuff like that, actually reflecting on it I mean it was as clear as day that I had dyslexia, but it was never really diagnosed so yeah it was quite funny looking back at it. (Bella)

**FIGURE 2:**
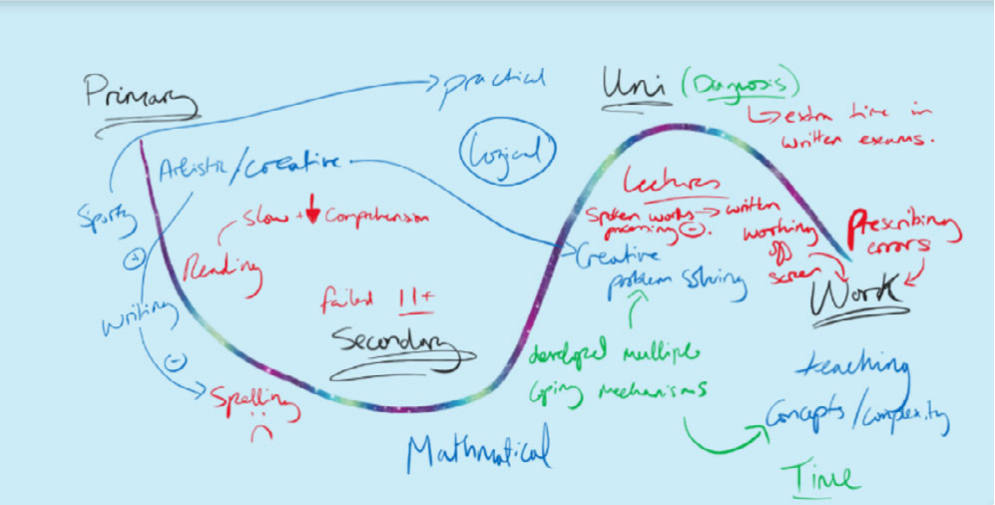
Bella's map. [Color figure can be viewed at wileyonlinelibrary.com]

The mapping made abstract concepts like transitions from university to clinical teaching, professional identity and resilience, easier to explore and discuss (e.g. Figure 2). The time taken to reflect, guided by the interview topic sheet, and creating a visual representation of their journeys was a cathartic experience for some:

I've never really thought about how it affected my whole journey … I found it quite interesting to reflect on my pre and post diagnoses experiences. (Rachel)

The time provided to reflect and create their maps, with the aid of the interview topic questions, mitigated the potential issues with working memory and processing speed. This reduced cognitive load and the potential stress that could have been induced through traditional interviews.^33^

2 ‘I can see patterns very quickly’—The visual nature of mapping is a key advantage.

The visual nature of the maps provided additional layers of meaning such as metaphors and spatial organisation, opening interpretive possibilities which may not be present in wholly verbal interviews. Being able to look at their maps aided verbal articulation to communicate their experiences. Creating maps in their own way coincided with the ways in which participants described how dyslexia shaped the way they think and communicate:

I've always found pattern recognition a real strength of mine. (Bob)

I have a different way of seeing things. (Blanche)

**FIGURE 3:**
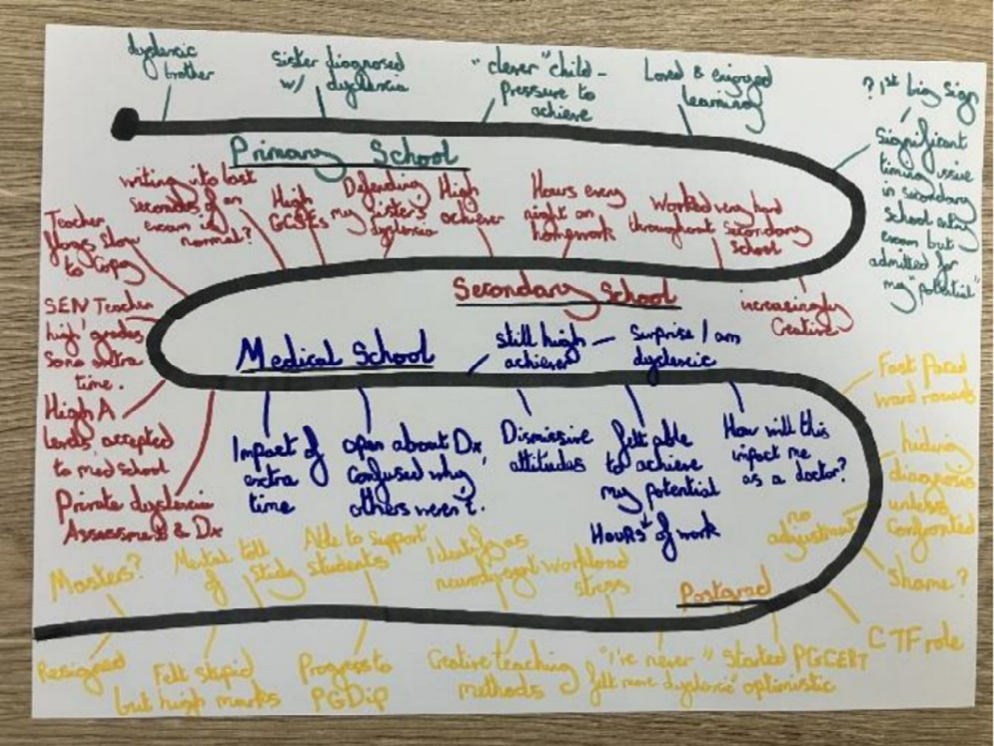
Rachel's map. [Color figure can be viewed at wileyonlinelibrary.com]

When describing her map, Rachel explained:

The colours aren't relevant … I just like colour … I kind of literally wanted to do a kind of winding map of the kind of main part of my education, I suppose … like my teaching, I take more creative approaches. (See Figure 3)

Despite the positive aspects, having dyslexia can mean individuals struggle with ‘expressing myself in words and in writing’ (Bob), word retrieval and verbal articulation:

I will know it, but the right words won't come out of my mouth, but they are in my head … so frustrating. (Gail)

I think medicine is built around people being very eloquently spoken, like incredibly articulate … . sometimes I know it, but I cannot say the precise words you want me to say. That doesn't mean that that's not what's going through my brain. (Bella)

The maps therefore supported articulation of reflections and aided explanations.

Having the freedom to express their stories and create maps in a mode that reflects how they best mediate information contributed to an autonomous approach that aligned with their cognitive strengths^6^:

I struggle being neat and tidy in terms of handwriting and things like that … I can see patterns very quickly. (Blanche)

This encouraged a sense of ownership and agency over the storytelling process, enabling them to shape the narrative in ways that felt meaningful and manageable to them. This further evidences the need for methods to enable participants to be engaged in their creation of data and enable participant authority in the research process.^18^, ^19^

3 Clear instructions for participation are vital

Providing clear instructions was vital. Blanche recalled making ‘three different versions of it [map]’ feeling that her map needed to be easily translated by the researcher:

I think the first one was quite a bit like a spider diagram and then I sort of thought I can understand that but maybe you won't understand the sort of mishmash of things. (Blanche)

In hindsight, participants needed clearer reassurance that their maps did not need to be ‘perfect’ and were simply prompts for storytelling, not for researcher‐led interpretation. To avoid interpretive ambiguity, we coded interview transcripts based on participants' reflections, not the maps themselves.

Providing the interview prompts before the interview meant they could prepare their responses and organise their stories visually. This was another strategy that was particularly helpful in relation to working memory and processing speed, as Blanche explained:

I think I struggle a lot with auditory things if I'm given a lot of verbal instructions … I don't know whether I just switch off or whether I just don't have the short term memory to retain a variety of verbal instructions … .I find it quite hard, especially under pressure in a time sort of thing … to quickly respond to something. It can be quite hard.

This reduced the pressure on their verbal fluency. Their maps provided visual and spatial prompts which supported memory retrieval and sequencing:

So, I don't know if this is a dyslexia thing or a personality thing, but for me I like that things make sense and be in order. So, for me this is a chronological list really. And for me that's what makes most sense because it's built like building blocks on what happens next. (Gail)

**FIGURE 4:**
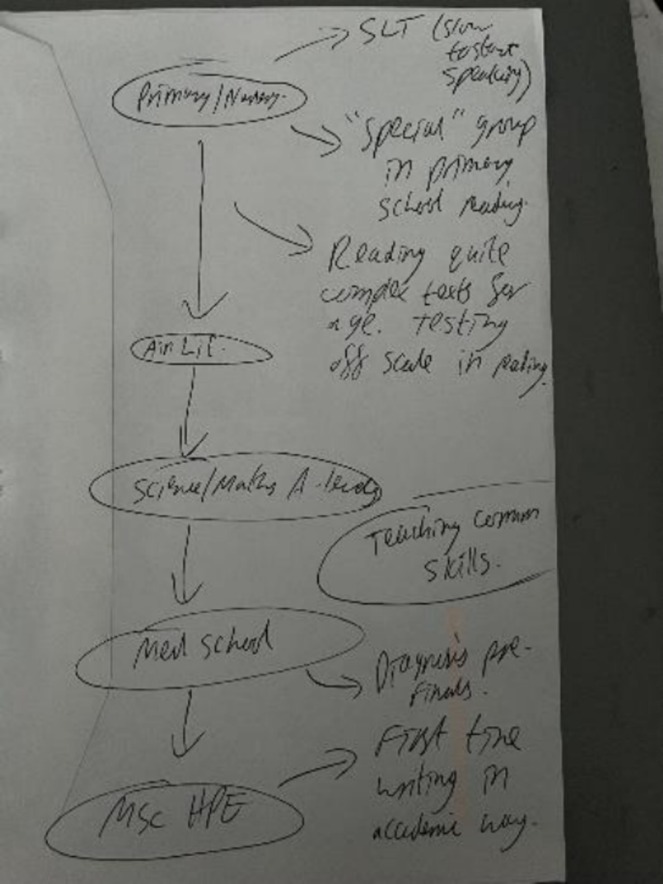
Bob's map. [Color figure can be viewed at wileyonlinelibrary.com]

The maps therefore presented visual scaffolding, or ‘building blocks’ (e.g. see Bob's map, Figure 4), enabling participants to frame their journeys in personally meaningful ways.

Example templates could have been provided; however, this may have inhibited their creativity—a key asset of dyslexia.^7^ Moreover, the openness of the task offered the potential to yield more authentic and diverse representations. Despite this, the open‐ended nature of journey mapping may result in very different creations, and although this diversity reflects individual expressions, it could complicate comparative analysis and thematic coding across the data set. Creating the maps also takes time,

I went back to it a few times as I remembered things and wanted to add them. (Peter)

This may be burdensome for some participants or deter potential participants from taking part. The time commitment must be clear to potential participants so that they are fully informed before agreeing to partake.

4 Mapping can elicit significant emotions.

The maps helped elicit memories in depth and with thick descriptions. Participants ‘saw’ how far they had come along their journeys, and what they had achieved:

It made me think actually about the different points in my education where I have struggled and stuff and it made me think, yeah, I have preserved a lot of be able to get where I am. (Blanche)

Participating in the research therefore helped validate their hard work and evidenced how dyslexia should not mean that individuals cannot achieve in education. It did however make learning difficult, ‘it's stopped me doing things’ (Bob) and their journeys complicated:

I find lectures really difficult … my real weakness … I probably wasn't the best student. I didn't go to a lot of lectures because of that reason. (Bella)

Every year I would get the same detention from the same English teachers because they would do this yearly English test, yearly spelling test and I would always come like dead last in the class …. (Peter)

I didn't get in [to university] the first time … so I was just working in a in a factory, and I didn't really have money to go … it's like £300 or whatever it is to get diagnosed as dyslexic …. (Peter)

The depth of reflection generated before and during the interview meant some negative experiences were recalled, leading to an emotional response:

Lots of things came up that were quite negative, like remembering challenges or difficulties around things that I've struggled with. (Gail)

It emphasised the affecting impact having dyslexia has on individuals, and their families:

I hadn't thought about it as much really as kind of a long term thing. I had a chat with my family saying that I was doing this and my mum was quite upset overall in that she thought that when I sort of put everything chronologically, she felt she probably should have realised when I was a kid that this was going on. (Peter)

Although, some found this useful to revisit their difficult times:

I think it definitely bought up some things I hadn't realised especially in my post graduate experience about how I felt about my dyslexia and how that's actually changed over time in several ways …up and down with how I feel about it, so it was useful in some ways. (Rachel)

To reduce psychological risk, participants received a debrief sheet with links to support services.^34^ Researchers must consider the emotional impact of sensitive topics and ensure robust ethical procedures are followed.

### 3.1 Limitations

Although our approach considered the inclusivity of the data creation, it is not without limitations. To be fully inclusive the research could have involved participants in the research process itself as co‐researchers.^35^ This principle was not fully actualised in this project as although the principal researcher was dyslexic, the participants recruited for the study were not recruited as co‐researchers. Although journey mapping aligns well with the cognitive strengths associated with dyslexia, it may not be universally suitable for all neurodivergent individuals or learning styles. Researchers should be mindful that no single method will accommodate every participant, and flexibility in methodological design is essential to uphold inclusivity.

## 4 CONCLUSION

The study contributes to inclusive research practice and addresses a key gap—exploring the experiences of health professionals with dyslexia not only as learners, but as educators. Journey mapping presents an inclusive, creative research tool that aligns with the cognitive strengths of participants with dyslexia. By foregrounding agency, accessibility and flexibility, we posit it offers richer participation and deeper data collection, compared to purely verbal interviews. This paper calls for researchers to consider the value of cognitive diversity, advocating methods that support neurodivergent ways of thinking and communicating. By creating space for their stories—through methods aligning to their cognitive strengths rather than highlighting their challenges—we shift the culture of research from doing research *on* to doing research *with* participants, promoting ethical and authentic engagement.

## AUTHOR CONTRIBUTIONS

We confirm that this manuscript is original, has not been published before and is not currently being considered for publication elsewhere. Sarah McLaughlin was the principal investigator for the research project, conducted all interviews, jointly analysed all the data and wrote the majority of this paper. Asim Ali contributed to the design of the project, analysed some data alongside Sarah McLaughlin and Steve Jennings and contributed to the revision and approval of the final article. Steve Jennings contributed to the design of the project, analysed all the data alongside Sarah McLaughlin and Asim Ali and contributed to the drafting, revision and approval of the final article.

## CONFLICT OF INTEREST STATEMENT

The authors have no conflicts of interest to declare.

## ETHICS STATEMENT

Ethical approval was granted by the University of Bristol Research Ethics Committee.

## Data Availability

The data that support the findings of this study are available on request from the corresponding author. The data are not publicly available due to privacy or ethical restrictions.

## References

[1] George RE , Sidhu M . Promoting inclusivity in health professions education. Clin Teach. 2023;20(6):e13606. doi:10.1111/tct.13606 37475641

[2] Denzin N , Lincoln Y . Handbook of Qualitative Research. 3rded. Sage; 2005.

[3] Kahlke R , Maggio LA , Lee MC , et al. When words fail us: an integrative review of innovative elicitation techniques for qualitative interviews. Med Educ. 2025;59(4):382‐394. doi:10.1111/medu.15555 39412120 PMC11906277

[4] McFarland B , Bryant L , Wark S , Morales‐Boyce T . Adaptive interviewing for the inclusion of people with intellectual disability in qualitative research. J Appl Res Intellect Disabil. 2024;37(1):e13182. doi:10.1111/jar.13182 38044591

[5] National Health Service (NHS) . (2022). Dyslexia: Overview. Available: https://www.nhs.uk/conditions/dyslexia/

[6] Made By Dyslexia (MBD) . (2024) Intelligence 5.0 Report. Available: https://www.madebydyslexia.org/resources/

[7] British Dyslexia Association (BDA) . (2025) What is dyslexia? https://www.bdadyslexia.org.uk/dyslexia/about-dyslexia/what-is-dyslexia

[8] Smith‐Spark JH , Fisk JE . Working memory functioning in developmental dyslexia. Memory. 2007;15(1):34‐56. doi:10.1080/09658210601043384 17479923

[9] Stenneken P , Egetemeir J , Schulte‐Körne G , Müller HJ , Schneider WX , Finke K . Slow perceptual processing at the core of developmental dyslexia: a parameter‐based assessment of visual attention. Neuropsychologia. 2011;49(12):3454‐3465. doi:10.1016/j.neuropsychologia.2011.08.021 21903119

[10] Nind M . The practical wisdom of inclusive research. Qual Res. 2017;17(3):278‐288.

[11] Tarafdar SA , Seoudi N , Luo R , Winston K . Experiences of medical students and doctors with dyslexia: a systematic review. Medical Education; 2025. 10.1111/medu.15615 PMC12242890 39936482

[12] Ryder J , Norwich B . Dyslexia in Higher Education: Advances in Theory and Practice. Routledge; 2018.

[13] National Health Service (NHS) . (2025b) Neurodiversity Support. https://london.hee.nhs.uk/professional-development/dyslexia

[14] National Health Service (NHS) . (2025a) Dyslexia—Guide for managers and colleagues. Available—Dyslexia, Dyscalculia, Dyspraxia, and other Neuro Developmental Conditions | Nottinghamshire Healthcare NHS Foundation Trust

[15] Murphy A , Stevenson J . Occupational potential and possible selves of master's level healthcare students with dyslexia: a narrative inquiry. J Occup Sci. 2019;26(1):18‐28. doi:10.1080/14427591.2018.1517387

[16] Walmsley J , Strnadová I , Johnson K . The added value of inclusive research. J Appl Res Intellect Disabil. 2018;31(5):751‐759. doi:10.1111/jar.12431 29231273

[17] Nind M . What is inclusive research? Bloomsbury Academic; 2014.

[18] Walmsley J , Johnson K . Inclusive Research With People With Learning Disabilities Past, Present and Futures. Jessica Kingsley; 2003.

[19] Bigby C , Frawley P , Ramcharan P . Conceptualizing inclusive research with people with intellectual disability. J Appl Res Intellect Disabil. 2014;27(1):3‐12. doi:10.1111/jar.12083 24390972

[20] Kara H . Creative Research Methods in the Social Sciences: A Practical Guide. Policy Press; 2015. doi:10.56687/9781447320258

[21] Nind M , Vinha H . Creative interactions with data: using visual and metaphorical devices in repeated focus groups. Qualitat Res. 2016;16(1):9‐26. doi:10.1177/1468794114557993 PMC4725156 26865833

[22] Kearney KS , Hyle AE . Drawing out emotions: the use of participant‐produced drawings in qualitative inquiry. Qualitat Res. 2004;4(3):361‐382. doi:10.1177/1468794104047234

[23] McLaughlin S . Using Bourdieu’s concept of habitus to explore higher education decision‐making for working class women on an access to higher education course. Stud Ed Adults. 2024;1‐19.

[24] Kolar K , Ahmad F , Chan L , Erickson PG . Timeline mapping in qualitative interviews: a study of resilience with marginalized groups. Int J Qual Methods. 2015;14(3):13‐32. doi:10.1177/160940691501400302

[25] Patterson M , Markey M , Somers J . Multiple paths to just ends: using narrative interviews and timelines to explore health equity and homelessness. Int J Qual Methods. 2012;11(2):132‐151. doi:10.1177/160940691201100202

[26] Berends L . Embracing the visual: using timelines with in‐depth interviews on substance use and treatment. Qual Rep. 2011;16:1‐9.

[27] Moon‐Seo SK , Campos M , Munsell SE . Exploring college students' experiences drawing a journey map. Educ Res: Theory Practice. 2023;34(2):57‐61.

[28] Nielsen AMW , Bruselius‐Jensen M . Journey mapping as a method to make sense of participation. In: Bruselius‐Jensen M , Pitti I , Tisdall EKM , eds. Young People's Participation: Revisiting Youth and Inequalities in Europe. Bristol University Press; 2021:235‐254.

[29] Blumer H . The methodological position of symbolic interactionism. Symbolic Interactionism: Perspective and Method. 1969;1:60.

[30] Mead GH . Mind, Self, and Society From the Perspective of a Social Behaviourist. University of Chicago; 1934.

[31] Rainford J . Confidence and the effectiveness of creative methods in qualitative interviews with adults. Int J Soc Res Methodol. 2020;23(1):109‐122. doi:10.1080/13645579.2019.1672287

[32] Braun V , Clarke, V . Thematic analysis: a practical guide. Sage; 2022.

[33] Chaisson NF , Ashton RW . Virtual interviews and their effect on cognitive load for graduate medical education applicants and programs. ATS scholar. 2021;2(3):309‐316. doi:10.34197/ats-scholar.2020-0156PS 34667981 PMC8518638

[34] Brenner ME . Interviewing in educational research. In: Handbook of Complementary Methods in Education Research. Routledge; 2012:357‐370.

[35] Wolfensberger W . The definition of normalization: update, problems disagreements and misunderstandings. In: Flynn R , Bitsch K , eds. Normalisation, Social Integration and Community Services. University Park Press; 1980:71‐115.

